# ICTV Virus Taxonomy Profile: Hypoviridae 2023

**DOI:** 10.1099/jgv.0.001848

**Published:** 2023-05-16

**Authors:** Sotaro Chiba, Leonardo Velasco, María A. Ayllón, Nobuhiro Suzuki, Shin-Yi Lee-Marzano, Lying Sun, Sead Sabanadzovic, Massimo Turina

**Affiliations:** 1 Graduate School of Bioagricultural Sciences, Nagoya University, Furo-cho, Chikusa-ku, Nagoya 464-0861, Japan; 2 Instituto Andaluz de Investigación y Formación Agraria, Centro de Málaga, Almería, 290140 Malaga, Spain; 3 Centro de Biotecnología y Genómica de Plantas (UPM-INIA), Universidad Politécnica de Madrid Campus de Montegancedo Pozuelo de Alarcón, Madrid 28223, Spain; 4 Departamento de Biotecnología-Biología Vegetal, ETSI. Agronómica, Alimentaria y de Biosistemas, Universidad Politécnica de Madrid, Madrid 28040, Spain; 5 Institute of Plant Science and Resources, Okayama University, Kurashiki 710-0046, Japan; 6 Department of Agriculture, Agricultural Research Service, Application Technology Research Unit, Toledo, OH 43606, USA; 7 College of Plant Protection, Northwest A&F University, Taicheng Road 3#, Yangling, Shaanxi, 712100, PR China; 8 Department of Biochemistry, Molecular Biology, Entomology and Plant Pathology, Mississippi State University, Mississippi State, MS 39762, USA; 9 Institute for Sustainable Plant Protection-CNR, Torino 10135, Italy

**Keywords:** ICTV Report, Taxonomy, *Hypoviridae*

## Abstract

*Hypoviridae* is a family of capsidless viruses with positive-sense RNA genomes of 7.3–18.3 kb that possess either a single large open reading frame (ORF) or two ORFs. The ORFs appear to be translated from genomic RNA by non-canonical mechanisms, i.e. internal ribosome entry site- and stop/restart translation. This family includes the genera *Alphahypovirus*, *Betahypovirus*, *Gammahypovirus*, *Deltahypovirus*, *Epsilonhypovirus*, *Zetahypovirus*, *Thetahypovirus* and *Etahypovirus*. Hypovirids have been detected in ascomycetous and basidiomycetous filamentous fungi and are considered to replicate in host, Golgi apparatus-derived, lipid vesicles that contain virus dsRNA as the replicative form. Some hypovirids induce hypovirulence to host fungi, while others do not. This is a summary of the ICTV report on the family *Hypoviridae*, which is available at www.ictv.global/report/hypoviridae.

## Virion

 No true virions are associated with members of the family *Hypoviridae*. Pleomorphic vesicles 50–80 nm in diameter [1], devoid of any detectable viral structural proteins but containing replicative form dsRNA and polymerase activity [2], are the only virus-associated structures that can be isolated from infected fungal tissue (Table 1); isolated vesicles co-purify with trans-Golgi apparatus markers [3].

**Table 1.** Characteristics of members of the family *Hypoviridae*

Example:	Cryphonectria hypovirus 1 strain EP713 (M57938), species *Alphahypovirus cryphonectriae*, genus *Alphahypovirus*
Virion	Capsidless (no true virions)
Genome	7.3–18.3 kb of linear, positive-sense, unsegmented RNA
Replication	Replication and transcription occur cytoplasmically, and for Cryphonectria hypovirus 1 in Golgi apparatus-derived membranous vesicles
Translation	Directly from genomic RNA containing a possible internal ribosomal entry site at the 5′-non-coding region. Cryphonectria hypovirus 1 ORF B is translated through re-initiation after the ORF A stop codon
Host range	Fungi; viruses identified in animals (arthropods) need confirmatory evidence
Taxonomy	Realm *Riboviria,* kingdom *Orthornavirae,* phylum *Pisuviricota*, class *Duploviricetes*, order *Durnavirales; *>7 genera and >35 species

## Genome

**Fig. 1.**
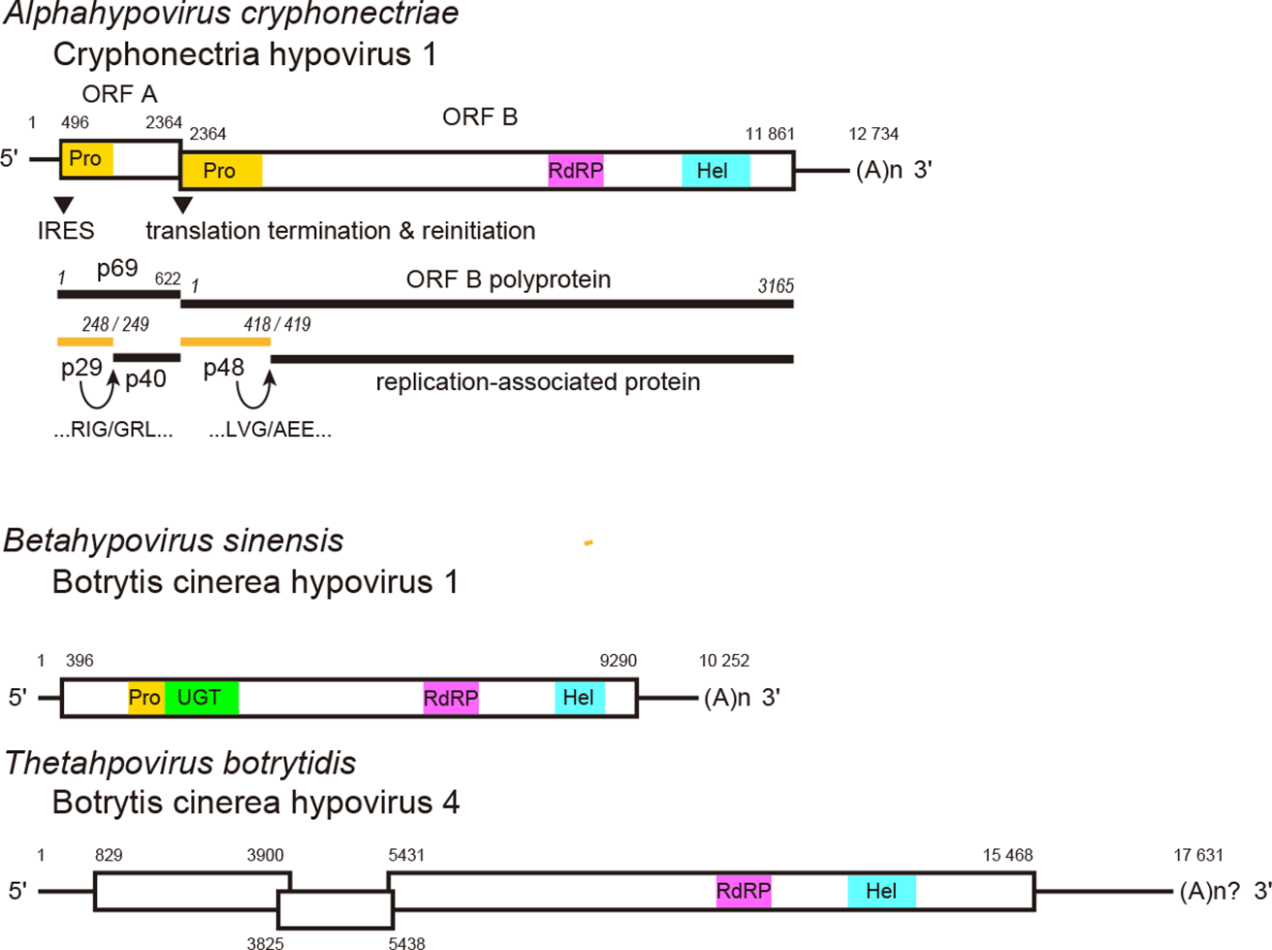
Genome organization of representative members of the family *Hypoviridae*. Arrows represent known sites of autoproteolysis. The abbreviations RdRP, Hel and UGT refer to the RNA-directed RNA polymerase, RNA helicase and UDP-glucose/sterol glucosyltransferase domains, respectively. IRES, internal ribosomal entry site; Pro, protease.

Hypovirus genomes range from 7.3 to 18.3 kb excluding a 3′-poly(A) tail of 20–30 nt, and possess one or two ORFs (Fig. 1) [4] flanked by relatively long 5′- and 3′-terminal non-coding regions (NCRs). Translational initiation for the first ORF on the genomic RNA is mediated by an internal ribosome entry site in the 5′-NCR extending to the coding domain in the case of Cryphonectria hypovirus 1. For hypovirids with a two ORF genome organization, a stop/restart mechanism is involved in the translation of the downstream ORF in which the pentanucleotide UAAUG, plays a critical role [5]. Many hypovirids have shorter-than-full-length, internally-deleted, defective interfering and defective replicative form dsRNA molecules; others have replicative forms of satellite-like RNAs [6,7]. The host RNA silencing pathway has been reported to promote defective interfering RNA production [8]. No function has been ascribed to any ancillary RNA.

## Replication

Positive- and negative-sense viral RNA synthesis is believed to occur cytoplasmically in host-derived lipid vesicles that contain linear dsRNA. The polymerase associated with vesicles transcribes ssRNA molecules *in vitro* that correspond in size to full-length dsRNA. Approximately 80 % of the polymerase products *in vitro* are of positive-sense. Except for the p50 of Cryphonectria hypovirus 2, hypovirid proteins are synthesized as part of a polyprotein that is autocatalytically cleaved by viral proteases such as p29 and p48 (Cryphonectria hypovirus 1) and p52 (Cryphonectria hypovirus 2). Smaller proteins encoded by the 3′-proximal ORF of Cryphonectria hypovirus 1 have been identified in the vesicle-associated polymerase complex, suggesting extensive processing of the ORF B-encoded polyprotein *in vivo* by unknown viral or host proteases [2]. Cryphonectria hypovirus 1 p29 enhances virus replication *in cis* and *in trans*, possibly by suppressing antiviral RNA silencing [8]. The p48 protein encoded by Cryphonectria hypovirus 1 ORF B is required for initiation, but not maintenance of viral RNA replication [9].

## Taxonomy

Current taxonomy: ictv.global/taxonomy. The family *Hypoviridae* includes the genera *Alphahypovirus*, *Betahypovirus*, *Gammahypovirus*, *Deltahypovirus*, *Epsilonhypovirus*, *Zetahypovirus*, *Thetahypovirus* and *Etahypovirus*.

## Resources

Full ICTV Report on the family *Hypoviridae*: www.ictv.global/report/hypoviridae.
